# ADULTS' VIEWS OF AGING AS AN UNDERESTIMATED RISK FACTOR FOR HEALTH, WELL-BEING, AND LONGEVITY

**DOI:** 10.1093/geroni/igac059.1674

**Published:** 2022-12-20

**Authors:** Manfred Diehl, Susanne Wurm, Becca Levy

## Abstract

Worldwide population aging has greatly increased the diversity of the “aging enterprise.” Research has established a solid portfolio of evidence showing that positive and negative views of aging represent independent resilience or risk factors for health, well-being, and longevity. Indeed, the effects of views of aging remain significant beyond the effects of other risk factors for health and mortality. This raises the following questions: What do we currently know about the effect of self-perceptions of aging (SPA) and subjective age (SA) on health, well-being, and longevity? What are recent advancements and perspectives? Which research questions should be addressed to stimulate further, sustainable developments in research and practice? This symposium addresses these questions with a diverse set of presentations and from different perspectives. Wahl and colleagues will discuss the role of SPA in the clinical context, namely in a sample of older adults with terminal cancer comparing them to older adults without a terminal illness. Based on a population-based sample, Wurm and Schaefer will report findings on the impact of different gain- and loss-related SPA and SA on mortality over a 23-year period. Building on an earlier meta-analysis, Westerberg and colleagues evaluated data from over 100 studies and will present the findings of a systematic review on the role of SPA and SA for health and longevity. Finally, Nehrkorn-Bailey et al. will present findings from a clinical trial that addressed views of aging as a mechanism to promote physical activity. Dr. Becca Levy will serve as the discussant.

